# Enhanced Mucosal Immune Responses Induced by a Combined Candidate Mucosal Vaccine Based on Hepatitis A Virus and Hepatitis E Virus Structural Proteins Linked to Tuftsin

**DOI:** 10.1371/journal.pone.0123400

**Published:** 2015-04-13

**Authors:** Yan Gao, Qiudong Su, Yao Yi, Zhiyuan Jia, Hao Wang, Xuexin Lu, Feng Qiu, Shengli Bi

**Affiliations:** National Institute for Viral Disease Control and Prevention, Chinese Center for Disease Control and Prevention, Beijing, China; Harvard Medical School, UNITED STATES

## Abstract

Hepatitis A virus (HAV) and Hepatitis E virus (HEV) are the most common causes of infectious hepatitis. These viruses are spread largely by the fecal-oral route and lead to clinically important disease in developing countries. To evaluate the potential of targeting hepatitis A and E infection simultaneously, a combined mucosal candidate vaccine was developed with the partial open reading frame 2 (ORF2) sequence (aa 368–607) of HEV (HE-ORF2) and partial virus protein 1 (VP1) sequence (aa 1–198) of HAV (HA-VP1), which included the viral neutralization epitopes. Tuftsin is an immunostimulatory peptide which can enhance the immunogenicity of a protein by targeting it to macrophages and dendritic cells. Here, we developed a novel combined protein vaccine by conjugating tuftsin to HE-ORF2 and HA-VP1 and used synthetic CpG oligodeoxynucleotides (ODNs) as the adjuvant. Subsequent experiments in BALB/c mice demonstrated that tuftsin enhanced the serum-specific IgG and IgA antibodies against HEV and HAV at the intestinal, vaginal and pulmonary interface when delivered intranasally. Moreover, mice from the intranasally immunized tuftsin group (HE-ORF2-tuftsin + HA-VP1-tuftsin + CpG) showed higher levels of IFN-γ-secreting splenocytes (Th1 response) and ratio of CD4^+^/CD8^+^ T cells than those of the no-tuftsin group (HE-ORF2 + HA-VP1 + CpG). Thus, the tuftsin group generated stronger humoral and cellular immune responses compared with the no-tuftsin group. Moreover, enhanced responses to the combined protein vaccine were obtained by intranasal immunization compared with intramuscular injection. By integrating HE-ORF2, HA-VP1 and tuftsin in a vaccine, this study validated an important concept for further development of a combined mucosal vaccine against hepatitis A and E infection.

## Introduction

Hepatitis E virus (HEV) and Hepatitis A virus (HAV) are causative agents of viral acute hepatitis known to be enterically transmitted. HAV, a small, non-enveloped, positive strand RNA virus, mainly infects children[1]. HEV is also a non-enveloped virus that contains a single-stranded, positive-sense RNA genome [2]. It is reported as a major cause of acute clinical hepatitis in parts of Asia and other places with poor sanitation [3]. Of the 6 billion worldwide population, nearly 5 billion have been exposed to HAV and about 2 billion to HEV [4]. Both HEV and HAV are transmitted via the fecal-oral route and share many similar clinical symptoms, fulminant forms and epidemiological features, causing considerable economic loss. Combining vaccines to induce effective protective immunity against two or more similar diseases is a prudent public health strategy. For example, a combined vaccine that can protect against both hepatitis A and B infections simultaneously is currently available. Use of the combined HAV/HBV vaccine, which contains 360 EL.U (ELISA units) of inactivated hepatitis A virus and 10 μg of recombinant hepatitis B antigen absorbed on aluminum phosphate, was demonstrated to result in high immunization coverage rates of individuals due to fewer required injections with the combined vaccine [5, 6]. A vaccine targeting two or more pathogens has many advantages such as decreased number of injections, simplified vaccination schedules and reduced cost of vaccination. However, no mucosal vaccine that can protect against hepatitis A and E at the same time is available. Thus, developing a mucosal combined vaccine would be beneficial as dual infections with HEV and HAV have been reported [7].

Attenuated and inactivated vaccines against HAV are available [8], and an effective HEV vaccine was licensed recently[9]. However, these vaccines delivered by intramuscular injection were shown to produce few secretory IgA antibodies which could block viral infection timely in the mucosa tract [10, 11]. In addition, intramuscular injections are relatively costly, less acceptable to children and difficult to administer. Mucosal immunizations, including intranasal, oral, rectal and vaginal routes of administration, are newer approaches in vaccine development. They are aimed towards mimicking the natural infection route to stimulate a strong mucosal immune response and protect against microbial invasion and colonization at mucous membranes while also generating a systemic antigen-specific immune response. Intranasal vaccination has been shown to induce effective mucosal immunity in the urinary tract, oral and nasal cavities and the vaginal mucosa [12]. Indeed, nasal-associated lymphoid tissue (NALT) showed an intact immune response in 1-year-old mice, with signs of immunosenescence observed only in mice older than 2 years [13]. These results suggested that intranasal vaccination of the 5 to 6-week-old mice chosen in the current study would induce an intact immune response. Until now, seven vaccines targeting five of the main enteric pathogens (poliomyelitis *Salmonella typhi*, *Vibrio cholerae*, influenza and rotavirus) have been routinely administered mucosally to humans and achieved good immune responses [14]. Therefore, mucosal vaccination conceivably can achieve enhanced protective immunity at the frontline of pathogenic infections and potentially overcome the limitations of intramuscularly injected vaccines.

HEV ORF2 encodes a 71-kDa protein that contains the neutralizing epitope and functions as capsomeres to form the viral capsid. Based on results of a clinical trial published recently, a vaccine based on the recombinant HEV ORF2 protein provided clear evidence of protection against the occurrence of HEV [4]. The HAV VP1 epitope lies between amino acids (aa) 1–221 [15], and several reports have indicated that vaccines based on this region of the VP1 protein generally achieved good immune effects [16]. In this study, a partial ORF2 sequence (aa 368–607) of HEV and partial VP1 sequence (aa 1–198) of HAV were used to construct a combined vaccine.

Tuftsin is a naturally occurring tetrapeptide (threonine-lysine-proline-arginine) derived from the Fc domain of the heavy chain of IgG [17]. It can be recognized by specific receptors on macrophages and microglia, which express tuftsin receptors, and is capable of targeting proteins to these sites. Tuftsin also acts as a stimulatory factor to enhance cellular processes such as chemotaxis, migration and antigen presentation [18]. Several studies have indicated that tuftsin conjugates could increase production of antigen-specific antibodies [19–21]. One study reported that tuftsin was not immunogenic itself, but it could strengthen the humoral immune response to the antigen to which it was linked [22].

In this work, we constructed a novel mucosal vaccine based on HE-ORF2 and HA-VP1. The two proteins were constructed by adding lysine linkages at their C-terminal ends with or without a tetrapeptide tuftsin molecule as a stem. The four recombinant proteins were purified by diethylaminoethyl (DEAE) chromatography. Through the intramuscular or intranasal routes, immunization with the combination of HEV-ORF2-tuftsin and HAV-VP1-tuftsin (plus CpG adjuvant) was compared with the combination of HEV-ORF2 and HAV-VP1 (plus CpG adjuvant) as the no-tuftsin control group. Enzyme-linked immunosorbent assays (ELISA), flow cytometry and enzyme-linked immunospot (ELISPOT) assays were performed to evaluate the humoral and mucosal immune responses, respectively.

## Materials and Methods

### Construction of recombinant protein expression plasmids

The synthetic HE-ORF2-tuftsin and HA-VP1-tuftsin antigen genes, encoding aa 368–607 of HE-ORF2 linked to tuftsin and aa 1–198 of HA-VP1 linked to tuftsin, respectively, were codon-optimized for expression in *Escherichia coli*. By PCR amplification using *Pfu* DNA polymerase (Promega, Madison, WI, USA), two genetic constructs were prepared for the expression of HE-ORF2 (aa 368–607) or HA-VP1 (aa 1–198) in *E*. *coli* without tuftsin as a control plasmid. The specific primers for HE-ORF2 synthesized by Sangon Biotech (Shanghai, China) were 5’-GGAATTCCATATGATCGCTCT-3’ (forward) and 5’-GGAATTCCATATGATCGCTCT-3’ (reverse). The specific primers for HA-VP1 were 5’-GGAATTCCATATGGTTGGTGACG-3’ (forward) and 5’-GGAATTCCATATGATCGCTCT-3’ (reverse). After an initial denaturation at 94°C for 5 min, all reactions were subjected to 35 cycles at 94°C for 30 s, 56°C for 30 s and 72°C for 45 s, followed by a final extension at 72°C for 5 min. After double-enzyme digestion with *Xho* I and *Nde* I, the products were cloned into the pET43a vector (Novagen, Billerica, MA, USA) and transformed into *E*. *coli* strain BL21 (DE3) (TransGen Biotech, Beijing, China). Ampicillin-resistant colonies were selected and identified by restriction endonuclease analysis of the plasmids as well as sequencing.

### Expression of recombinant protein expression plasmids

Freshly transformed *E*. *coli* BL21 (DE3) cells containing recombinant plasmids were inoculated into Luria-Bertani (LB) medium (10 g/l tryptone, 5 g/l yeast extract, 10 g/l NaCl) supplemented with 50 μg/ml ampicillin at 37°C. When the OD_600_ reached 0.6–0.8, expression was induced by adding isopropylthio-D-galactoside (IPTG) to a final concentration of 0.1 mM and incubated for an additional 4 h at 37°C. After centrifugation (4000 × *g*, 10 min, 4°C), the cell pellet was resuspended in lysate buffer (10 mM Tris-HCl, 0.5% Triton X-100, pH 8.0) and then sonicated. The total bacterial proteins, supernatant and inclusion bodies were separated by centrifugation (12,000 × *g*, 10 min, 4°C) and then subjected to 15% sodium dodecyl sulfate–polyacrylamide gel electrophoresis (SDS-PAGE) to assess protein expression. The four recombinant proteins were expressed using the same procedures and conditions as described above.

### Purification and renaturation of recombinant proteins

The four recombinant proteins were all purified by DEAE chromatography, followed by gel filtration chromatography and finally concentrated by ultrafiltration centrifugation (Millipore, Billerica, MA, USA). Fractions were sampled to analyze the protein distribution and assess the homogeneity by 15% SDS–PAGE. The samples were renatured by gradually removing the urea with PBS. Finally, the fractions including highly purified protein were concentrated by ultrafiltration centrifugation (3850 × *g*, 4°C). Furthermore, potential endotoxin lipopolysaccharide (LPS) in the purified protein solution was removed using the ToxinEraser Endotoxin Removal Kit (GenScript, Beijing, China). Residual LPS levels were determined using the ToxinSensor Chromogenic LAL Endotoxin Assay Kit (GenScript) according to manufacturer's instructions. The purified and renatured proteins were stored at 4°C.

### Identification of recombinant proteins by Western blot

Recombinant proteins were separated by 15% SDS-PAGE and transferred to polyvinylidene fluoride (PVDF) membranes (Bio-Rad, Hercules, CA, USA). After blocking the membranes for 2 h in 5% skim milk at 37°C, they were incubated with serum from a patient infected with HAV or HEV or a healthy volunteer (one of each type of serum was used), followed by the addition of alkaline phosphatase-conjugated goat anti-human IgG as the secondary antibody for Western blot analysis. Signals were detected on Hyperfilm ECL (Amersham, Buckinghamshire, UK).

### Mice and immunization

Specific-pathogen-free female Balb/c mice aged 5–6 weeks were obtained from Vital River (Beijing, China). This research was approved by the Experimental Animal Ethics Committee, Institute for Viral Disease Control and Prevention (permit number: 2013-06-R-033). All mice were maintained under specific pathogen-free conditions at the Laboratory Animal Center, Chinese Center for Disease Control and Prevention. Experimental protocols and housing conformed to the Chinese Regulations for the Administration of Affairs Concerning Experimental Animals. Forty mice were randomly assigned to five groups to receive vaccinations as follows: (1) 10 μg HE-ORF2-tuftsin + 10 μg HA-VP1-tuftsin + 10 μg CpG by intramuscular (IM-tuftsin) or intranasal (IN-tuftsin) immunization; (2) 10 μg HE-ORF2 + 10 μg HA-VP1 + 10 μg CpG by intramuscular (IM-no-tuftsin) or intranasal (IN-no-tuftsin) immunization; (3) PBS (intranasal). Every group was immunized three times on day 0, 15 and 30.

Blood samples and fresh fecal pellets were collected once every 14 days from day 1 to 45. Blood samples were centrifuged (5000 × *g*, 10 min, 4°C) and stored at −80°C until used for analysis. Approximately 200 mg of each feces sample was suspended in 600 μl PBS with 0.5% BSA and Protease Inhibitor Cocktail (Roche, Basel, Switzerland) and incubated overnight at 4°C. The suspension was centrifuged at 16,000 × *g* for 10 min at 4°C, and the supernatant was stored at −80°C. Vaginal, respiratory tract and small intestine secretions were flushed with PBS containing 0.5% BSA and Protease Inhibitor Cocktail. IgA in the supernatants was assayed by ELISA. Mice were sacrificed on day 45, and splenocytes were isolated to perform ELISPOT assays.

### IFN-γ ELISPOT assay

Numbers of IFN-γ producing cells were quantified with an ELISPOT kit (DAKEWE Biotech, Shenzhen, China). Cells from two spleens were pooled for a total of three samples per group of mice, which were sacrificed two weeks after the last immunization. Spleen cells (2.5 × 10^5^) were added to MultiScreen 96-well filtration plates (DAKEWE Biotech), which were precoated with an anti-mouse IFN-γ capture antibody, and cultured with 40 μg/ml of the corresponding protein or phorbol myristate acetate (PMA) (positive control) as an antigenic stimulator. The spots were counted with an automated ELISPOT reader.

### Detection of specific antibodies by ELISA

Serum IgG and IgA antibody responses were detected by ELISA. Flat transparent 96-well microtiter plates (Thermo Fisher, Waltham, MA, USA) were coated with the corresponding antigen at a concentration of 5 μg/mL in 100 μl per well overnight at 4°C. After blocking with 5% skim milk, serial 2-fold dilutions of samples (i.e., serum, fecal suspensions, vaginal, respiratory tract and small intestine secretions) were applied in duplicate wells and incubated for 2 h at 37°C. After washing with PBS with 0.05% Tween-20 (PBST, V/V), 100 μl of horseradish peroxidase (HRP)-conjugated goat anti-mouse IgG or anti-mouse IgA (α-chain specific; Sigma-Aldrich, St. Louis, MO, USA) was added and then incubated for 1.5 h at 37°C. One hundred microliters of the 3, 3’, 3, 5’-tetramethylbenzidine (TMB) substrate was then added, and the reaction was terminated with concentrated sulfuric acid. The absorbance was measured at 450 nm in a spectrophotometer (Thermo Fisher). The group of mice receiving no treatment was used as a negative control. The positive cut-off value was 2.1 times above the normal negative control. Antibody effective dose were determined by the maximum dilution yielding positive results.

### Flow cytometry

Proportions of CD4^+^ and CD8^+^ T cells in the mouse splenocytes were detected by flow cytometry. Briefly, spleen cells (1 × 10^6^/well) derived from each group were co-cultured for 5 h with 10 μg/ml of the corresponding antigen or PBS alone. After the cells were washed and centrifuged, they were incubated with 10 μl of a FITC-conjugated rat anti-mouse CD8 antibody (eBioscience, San Diego, CA, USA) for a further 10 min in the dark at 4°C. After washing once and centrifuging, a PE-conjugated rat anti-mouse CD4 antibody (eBioscience) was added in a 100 μl volume and incubated for 25 min. The splenocytes were then washed twice and resuspended in 500 μl of PBS for flow cytometric analysis using a FACS Canto (BD Biosciences, Franklin Lakes, NJ, USA).

### Statistical analysis

GraphPad Prism 5 software (GraphPad, Inc., San Diego, CA) was used to graph and evaluate statistically significant differences between all groups. Specific IgG and IgA antibody effective dose are expressed as geometric mean titers (GMT). One-way analysis of variance (ANOVA) followed by Bonferroni’s *post-hoc* test was used to compare CD4^+^/CD8^+^ T cell ratios and ELISPOT values, and Kruskal-Wallis ANOVA followed by Dunn’s *post-hoc* test was used to compare ELISA values. *P* values of <0.05 were considered to be statistically significant.

## Results

### Purification and identification of recombinant protein expression

**Fig 1:**
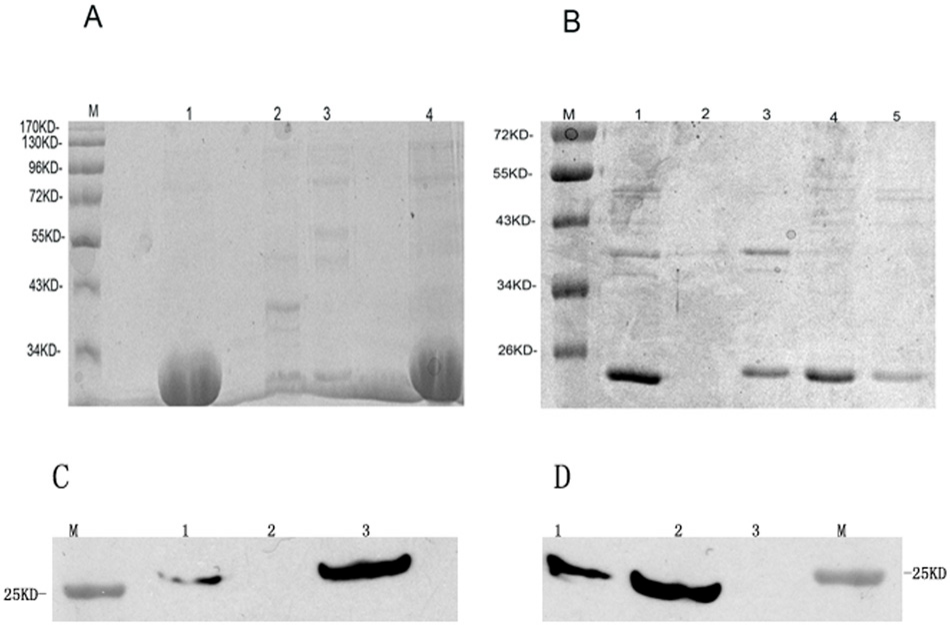
Purification and identification of recombinant proteins. Proteins were purified and identified by SDS-PAGE and Western blot analysis. (A) Lane M, marker; lane 1, HE-ORF2-tuftsin purified by DEAE chromatography; lanes 2 and 3, lysate of non-transformed BL21(DE3) cells; lane 4, HE-ORF2 purified by DEAE chromatography; (B) lane M, marker; lane 1, HA-VP1-tuftsin inclusion bodies dissolved with urea; lane 2, lysate of the non-transformed BL21(DE3) cells; lane 3, HA-VP1 inclusion bodies dissolved with urea; lane 4, HA-VP1-tuftsin purified by DEAE chromatography; lane 5, HA-VP1 purified by DEAE chromatography. (C) Western blot analysis of purified HE-ORF2-tuftsin and HE-ORF2. Lane M, marker; lane 1, HE-ORF2; lane 2, healthy serum control; lane 3, HE-ORF2-tuftsin. (D) Western blot analysis of purified HA-VP1-tuftsin and HA-VP1. Lane M, marker; lane 1, HA-VP1-tuftsin; lane 2, HA-VP1; lane 3, healthy serum control.

The four recombinant proteins, HE-ORF2-tuftsin, HE-ORF2, HA-VP1-tuftsin and HA-VP1, were all expressed within inclusion bodies, with a low amount of the proteins in the insoluble form after denaturation. Inclusion bodies were purified by DEAE chromatography. Most of the denatured proteins were dissolved in 100 mM NaCl solution and refolded by slow dialysis against buffers with decreasing urea concentrations. SDS-PAGE analysis demonstrated that the molecular weights of the HE-ORF2-tuftsin, HE-ORF2, HA-VP1-tuftsin and HA-VP1 proteins were at the expected sizes of approximately 28 kDa, 28 kDa (Fig 1A), 23 kDa and 23 kDa (Fig 1B), respectively. The purity was ~90% for HE-ORF2-tuftsin and HE-ORF2 or ~75% for HA-VP1-tuftsin and HA-VP1 as assessed by high performance liquid chromatography (HPLC). Concentrations of HE-ORF2-tuftsin, HE-ORF2, HA-VP1-tuftsin and HA-VP1 were 1.5, 2, 0.8 and 0.5 mg/ml, respectively, as determined with the BCA kit (Pierce, Rockford, IL, USA). Endotoxins were removed, and residual LPS levels were determined using commercial kits as described in Materials and methods. The concentrations of LPS detected in the four protein preparations were all ~5 EU/mL.

Western blot analysis using positive serum from patients infected with HAV and HEV further confirmed the identity of the four recombinant proteins. The HE-ORF2-tuftsin and HE-ORF2 proteins presented similar reactive bands at the molecular weight of 28 kDa (Fig 1C), and both HA-VP1-tuftsin and HA-VP1 showed a single band with the molecular weight of 23 kDa (Fig 1D). These values were consistent with the theoretical molecular weights, indicating that the recombinant proteins possessed good antigenicity.

### Recombinant proteins elicit antigen-specific IgG

**Fig 2:**
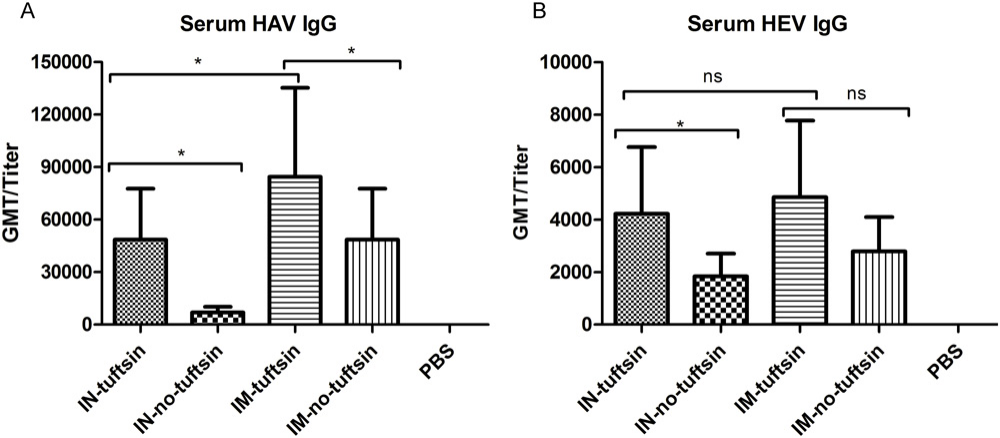
Profiles of total IgG production in humoral immune response to vaccination. (A) Anti-HAV IgG and (B) anti-HEV IgG were detected by indirect ELISA after immunization of the immunized groups as indicated. The IN-tuftsin group was intranasally administered HE-ORF2-tuftsin + HA-VP1-tuftsin + CpG. The IN-no-tuftsin group was intranasally administered HE-ORF2 + HA-VP1 + CpG. The IM-tuftsin group was intramuscularly administered HE-ORF2-tuftsin + HA-VP1-tuftsin + CpG. The IM-no-tuftsin group was intramuscularly administered HE-ORF2 + HA-VP1 + CpG. Each serum effective dose (presented as the GMT) is the reciprocal value of the maximum positive dilution. Error bars represent the standard error of the mean (SEM). *, *P*<0.05, **, *P*<0.01, ***, *P*<0.001; ns, no significant difference.

To evaluate whether tuftsin could enhance the level of antigen-specific IgG in mice compared with those of the no-tuftsin group and to determine which inoculation route would be more effective for a mucosal vaccine against mucosally-transmitted pathogens, blood samples were collected 2 weeks after the last immunization. HAV- and HEV-specific IgG antibodies were detected by ELISA using the corresponding coating antigen, and the effective dose were determined as the reciprocal of the maximum dilution of serum that produced a positive reading. Both the tuftsin group and no-tuftsin group displayed apparent immune responses, but the tuftsin group showed obvious advantages. After the last immunization, the HEV IgG effective dose in the IM-tuftsin group and IM-no-tuftsin group were 4850 and 2785, respectively, while those in the IN-tuftsin group and IN-no-tuftsin group were 4222 and 1873, respectively (Fig 2A). HAV IgG effective dose in the IM-tuftsin group and IM-no-tuftsin group were 84448 and 46502, respectively, while those in the IN-tuftsin group and IN-no-tuftsin group were 48502 and 6964, respectively (Fig 2B). The IgG effective dose, except the HEV IgG by intramuscular injection, of the IN-tuftsin group were significantly higher than that of the IN-no-tuftsin group (*P*<0.05) (Fig 2B). These results indicate that the tuftsin plus CpG adjuvant group could induce a greater humoral immune response with the intranasal route than with the intramuscular route. No significant difference in effective dose was observed between the IN-tuftsin and IM-tuftsin groups against HEV, while the antibody response against HAV in the IM-tuftsin group was higher than that of the IN-tuftsin group.

### Recombinant proteins induce mucosal IgA

**Fig 3:**
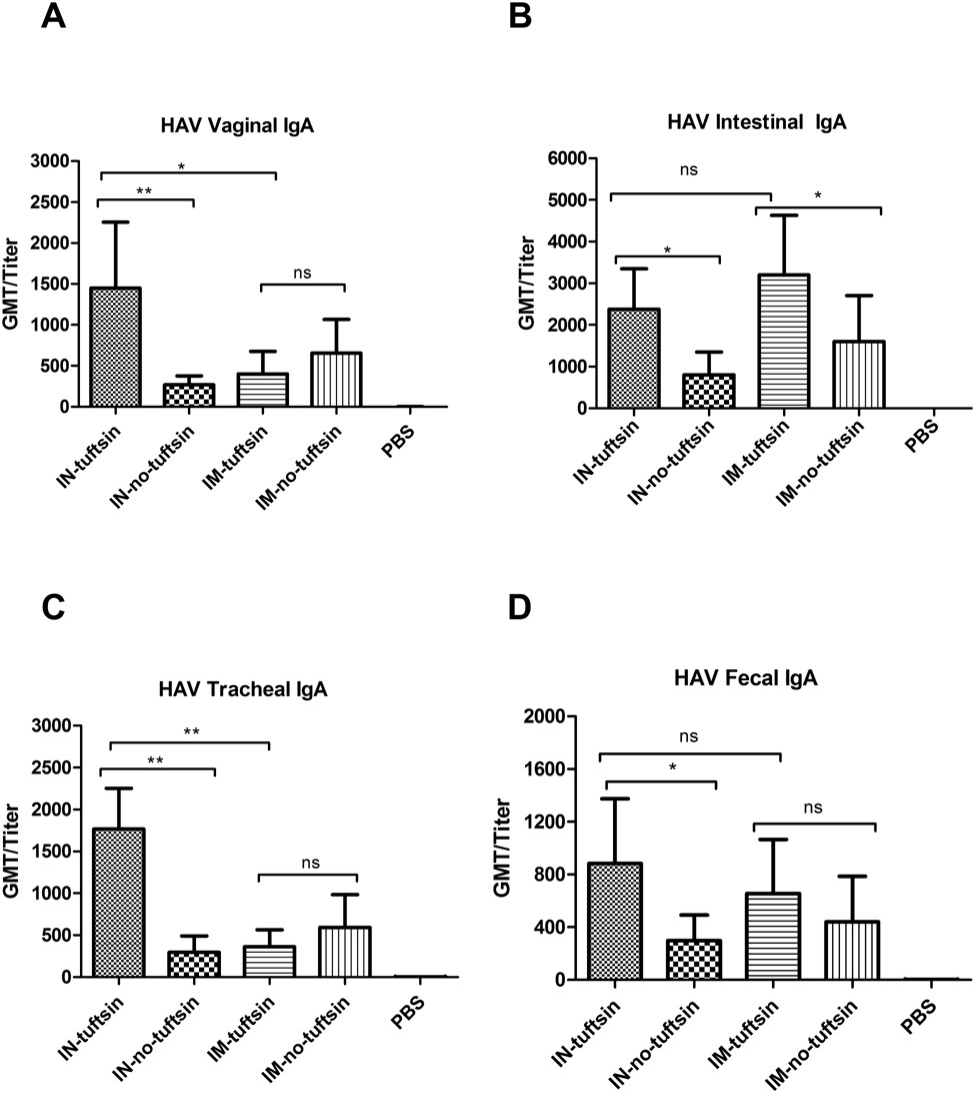
Profiles of HAV-specific IgA production in mucosal immune response to vaccination. HAV IgA was detected by indirect ELISA in (A) vaginal secretions, (B) intestine, (C) respiratory tract and (D) stool homogenates of the mouse groups immunized as indicated in Fig 2. Each serum effective dose (presented as the GMT) is the reciprocal value of the maximum positive dilution. Error bars represent the SEM. *, *P*<0.05, **, *P*<0.01, ***, *P*<0.001; ns, no significant difference.

**Fig 4:**
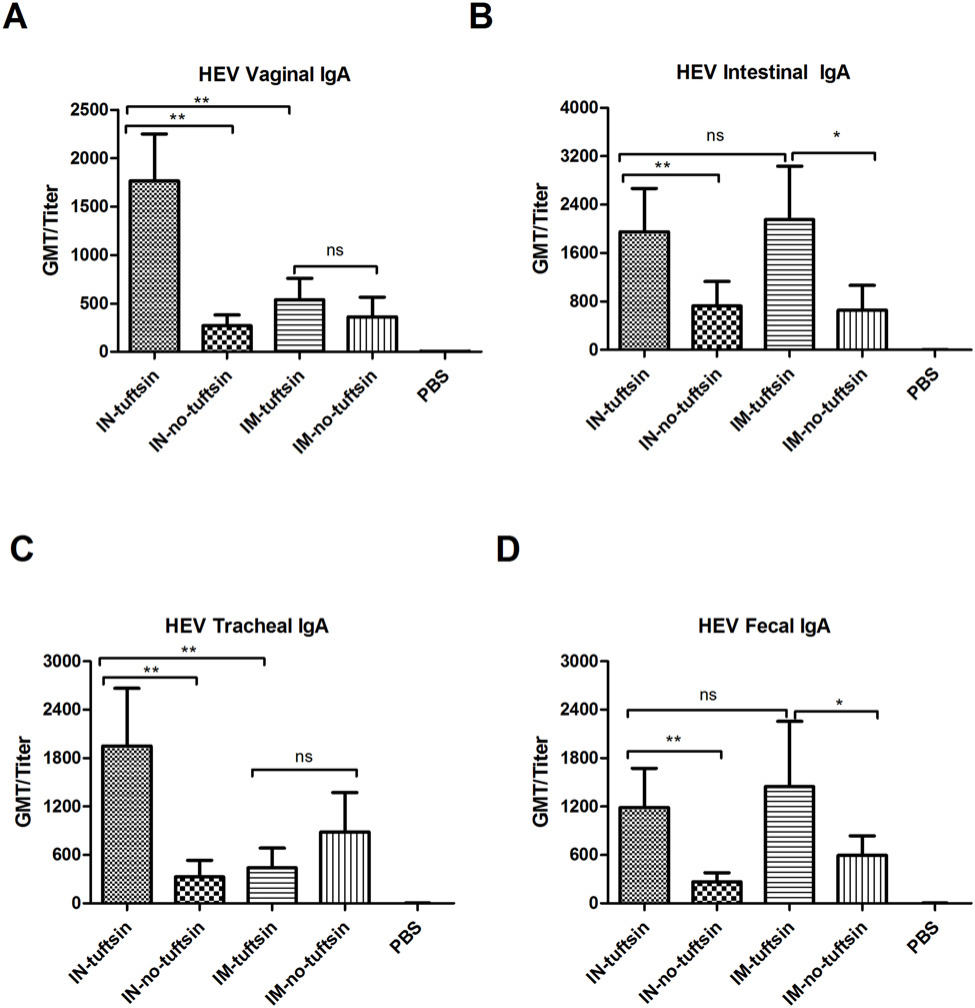
Profiles of HEV-specific IgA production in mucosal immune response to vaccination. HEV IgA was detected by indirect ELISA in (A) vaginal secretions, (B) intestine, (C) respiratory tract and (D) stool homogenates of the mouse groups immunized as indicated in Fig 2. Each serum effective dose (presented as the GMT) is the reciprocal value of the maximum positive dilution. Error bars represent the SEM. *, *P*<0.05, **, *P*<0.01, ***, *P*<0.001; ns, no significant difference.

To test whether higher levels of mucosal immune responses could be elicited in the tuftsin group than the no-tuftsin group and determine the more effective delivery route, samples of vaginal, small intestine and respiratory tract secretions, as well as feces suspension, were collected two weeks after the last inoculation to measure mucosal IgA antibody effective dose by ELISA. Differences in IgA effective dose against both HAV (Fig 3) and HEV (Fig 4) were apparent between the tuftsin group and no-tuftsin group with intranasal immunization at the vagina, intestine, respiratory tract and in the feces of vaccinated mice. The antibody levels of the tuftsin group and no-tuftsin group were also significantly different from the PBS negative control group. These results indicate that the tuftsin group showed a significantly higher level of mucosal immune response after intranasal inoculation. IgA effective dose elicited by intramuscular administration in mice were not as strong as those by the intranasal route, and no difference was observed between the tuftsin group and no-tuftsin group except at the gut.

### Induction of antigen-specific T cell responses

**Fig 5:**
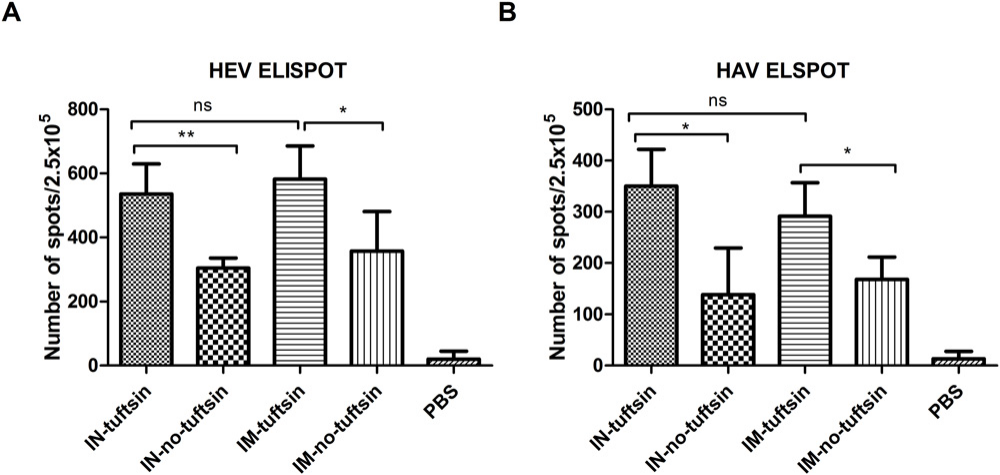
ELISPOT detection of *in vitro* IFN-γ production by splenocytes of vaccinated mice. Splenocytes from two spleens were pooled for a total of three samples per immunization group and analyzed by ELISPOT. (A) Numbers of spleen cells targeting HEV and (B) numbers of spleen cells targeting HAV were detected in the mouse groups immunized as indicated in Fig 2. Error bars represent the SEM. *, *P*<0.05, **, *P*<0.01, ***, *P*<0.001; ns, no significant difference.

To evaluate the Th1 cellular immune response in spleens of vaccinated mice, ELISPOT assays were performed to detect antigen-specific IFN-γ-producing T cells. Mouse spleen cells from each group were collected two weeks after the last immunization. As shown in Fig 5, the PBS group did not generate significant T cell responses, but antigen-specific IFN-γ producing cells were detected in all tuftsin and no-tuftsin animal groups. Moreover, the number of spots in the tuftsin group was significantly higher than that in the no-tuftsin group against HAV and HEV, but no statistically significant difference was observed between intranasal and intramuscular administration. These results provide evidence that HE-ORF2-tuftsin and HA-VP1-tuftsin plus a CpG adjuvant was effective in stimulating specific T cell responses.

### Relative proportions of CD4^+^ and CD8^+^ T cells after vaccination

**Fig 6:**
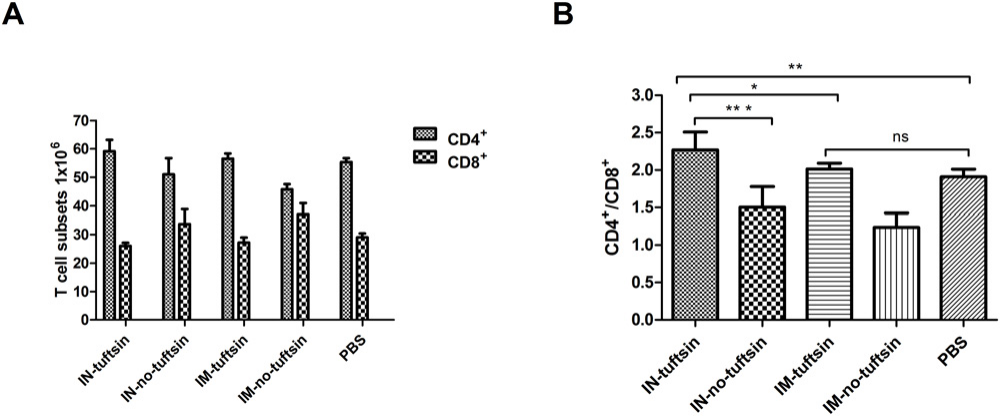
Proportions of CD4^+^ and CD8^+^ T cell subsets in splenotypes of vaccinated mice. (A) CD4^+^ T cell subsets and CD8+ T cell subsets of all groups. (B) CD4^+^/CD8^+^ T cell ratios of experimental groups and PBS control group. Mouse groups were immunized as indicated in Fig 2. Error bars represent the SEM. *, *P*<0.05, **, *P*<0.01, ***, *P*<0.001; ns, no significant difference.

Flow cytometry analysis indicated that in the IN-tuftsin group the numbers of CD4^+^ and CD8^+^ increased and decreased, respectively, compared to the PBS and IN-no-tuftsin groups. Similar results were obtained by intramuscular injection (Fig 6A). The proportions of CD4^+^ and CD8^+^ T-cell subsets were significantly higher in mice of the IN-tuftsin group than those of the IN-no-tuftsin group (*P*<0.001), IM-tuftsin group (*P*<0.05) and PBS group (*P*<0.01); moreover, the IM-tuftsin group and PBS group showed no statistically significant difference (Fig 6B). The tuftsin group displayed greater activation of CD4^+^ and lower activation of CD8^+^ than did the no-tuftsin group with intranasal administration.

## Discussion

HAV and HEV share many epidemiological and clinical features, as well as the same route of transmission. Hence, the development of a combined vaccine to prevent both viruses is highly desirable. Because exposures to HAV and HEV occur mostly at mucosal surfaces, targeting the mucosa to achieve protection is deemed a rational and efficient vaccination strategy. As no combined mucosal vaccine against HAV and HEV is available currently, we aimed to produce such a vaccine to prevent HAV and HEV simultaneously. In this study, HAV VP1 (aa 1–198) and HEV ORF2 (aa 368–607) were specifically selected to formulate the two-protein vaccine. VP1 (aa 1–198) appeared to be the dominant structural protein among the capsid proteins of HAV, and was demonstrated to be highly immunogenic previously [23]. HEV ORF2 (aa 368–607) naturally occurs as a homodimer and higher-order oligomers, and it was strongly recognized by HEV reactive sera in the oligomeric state. Furthermore, it was found to be more highly immunogenic compared with other viral peptides [24]. Because dissociated peptides have low antigenicity and immunogenicity, we linked tuftsin to the C-terminus of each of the two target proteins to enhance their targeting to the mucosa. Tuftsin contains only four amino acid residues, but it binds to specific receptors on the surfaces of polymorph-nuclear leukocytes and macrophages, mediating bactericidal and phagocytic activity. Conjugating tuftsin to four copies of M2e of influenza A has been reported to enhance the antigen-specific immune response [21]. Gokulan *et al*. also reported that linking tuftsin to envelope glycoproteins 41 (gp41) and 120 (gp120) of HIV enhanced the antigen-specific immune responses [19].

Eliciting immune responses by mucosal immunization usually relies on the co-administration of an appropriate adjuvant, which can initiate and maintain the transition from innate to adaptive immunity. In our study, we chose synthetic CpG oligonucleotides as an adjuvant to boost the immunogenicity of the target proteins. CpG-ODN containing unmethylated CpG has been reported to improve the immune response in co-administered vaccines. Many animal studies indicated that CpG-ODN can induce strong type 1 T cell responses in mice, thereby enhancing the protective efficacy of antiviral vaccines [25–27].

After inoculation of the two-protein combination vaccine, a difference in HEV IgG effective dose was only found between the IN-tuftsin and IN-no-tuftsin groups. However, the serum HAV-specific IgG effective dose in the tuftsin and no-tuftsin groups showed significant differences with both inoculation methods. The results indicated that the tuftsin group could generate stronger humoral immunity than the no-tuftsin group. Groups vaccinated with or without tuftsin via intramuscular injection developed higher HAV IgG effective dose than those by intranasal immunization, suggesting that the HAV antigens may be intrinsically limited at inducing mucosal immunity [28] or that the amount of available antigen was reduced due to a technical issue during delivery.

The mucosal surfaces of the respiratory, gastrointestinal and genitourinary tracts are the first lines of defense against the entry of microorganisms. Thus, mucosal immunization has garnered much attention recently. As the predominant antibody type at mucosal surfaces, IgA is thought to bind to pathogens which infect at those sites and cause systemic disease [18]. In our study, intranasal vaccination elicited specific IgA antibody responses at various mucosal surfaces. We detected IgA in fecal suspensions and lavage samples of the vagina, small intestine and respiratory tract. These results are not surprising since the different types of mucosal surfaces are interconnected, and immune cells induced at one mucosal tissue may extend their effector functions to another remote mucosal site. All groups that received intranasal immunizations showed certain levels of HEV IgA and HAV IgA, with significant differences between the IN-tuftsin and IN-no-tuftsin groups. The results also showed that the intramuscular injection could not induce IgA effective dose in the respiratory tracts and vagina as high as those by intranasal immunization, which may be the consequence of a low total amount of delivered antigen or a non-optimal length of tuftsin in the fusion protein. This study demonstrated that a vaccine based on tuftsin delivered via intranasal administration could elicit a significantly enhanced level of mucosal immunity over that by intramuscular injection.

CD4^+^ T cells regulate immune reactivity and cytokine secretions as well as augment the ability of B cells to produce antibodies [29], while CD8^+^ T cells cause direct cytolysis of infected target cells and mediate immune inhibition[30]. In our study, the number of CD4^+^ T cells increased, while CD8^+^ T cells decreased, causing the CD4^+^ /CD8^+^ T cell ratio to increase in the tuftsin group compared with the PBS and no-tuftsin groups with both intranasal and intramuscular inoculations. Reductions in CD4^+^ T cell counts have been associated with increased susceptibility to subsequent infections [31]. Thus, an antigen fused to tuftsin may promote stronger immune responses in mice by stimulating production of antibodies, improving CD4^+^ T cell proliferation and inhibiting CD8^+^ T cells.

Moreover, detection of the secreted IFN-γ levels from splenocytes of immunized mice indicated that the recombinant protein fused with tuftsin could stimulate specific T cell responses effectively, particularly Th1 cellular-mediated immunity. The improved cellular immune responses against HAV and HEV in the intranasal tuftsin group compared with the no-tuftsin group was likely related to the immunostimulatory effect of tuftsin [22]. In the ELISPOT assay, numbers of IFN-γ spot-forming cells in the intramuscular tuftsin and no-tuftsin groups were high, with no significant difference between them. The results suggest that tuftsin has a good ability to enhance the antigenicity of a protein via mucosal inoculation, but the targeting ability of tuftsin is reduced when the protein is injected into the musculature where fewer leukocytes and macrophages reside.

In summary, the efficacy and protective functions of immunization with the CpG + HE-ORF2-tuftsin + HA-VP1-tuftsin formulation were analyzed and evaluated using the BALB/C mouse model. The results demonstrated that the tuftsin gene could facilitate the induction of antigen-specific IgA antibodies in the intestine, feces, vagina and respiratory tract. Furthermore, it elicited systemic antigen-specific IgG antibodies responses and Th1 cellular immune responses. The cellular immune responses in the IN-tuftsin groups were greater than those in the IM-tuftsin groups. Thus, linking the immunostimulatory molecule tuftsin to targeted sequences could highly potentiate the immunogenicity of a protein antigen. This study provides a new strategy for the development of novel protein-based mucosal vaccines. Future experiments will be designed to explore the interactions between the two types of antigens in the combined HAV and HEV vaccine and determine whether different doses of the two antigens would have adverse effects on the immunogenicity.

## Supporting Information

S1 ARRIVE Checklist (PDF)

S1 Fig Original Western blot analysis of purified HE-ORF2-tuftsin and HE-ORF2. Lane M, marker; lane 1, HE-ORF2; lane 2, healthy serum control; lane 3, HE-ORF2-tuftsin.
(DOC)

S2 Fig Original Western blot analysis of purified HA-VP1-tuftsin and HA-VP1. Lane M, marker; lane 1 and 2, HA-VP1-tuftsin; lane 3, HA-VP1; lane 4, healthy serum control.
(DOC)
