# Supplementary figures and images for: Enhanced Mucosal Immune Responses Induced by a Combined Candidate Mucosal Vaccine Based on Hepatitis A Virus and Hepatitis E Virus Structural Proteins Linked to Tuftsin
Source: PLoS One. 2015 Apr 13;10(4):e0123400. doi: 10.1371/journal.pone.0123400 (PMC4395237; doi:10.1371/journal.pone.0123400)

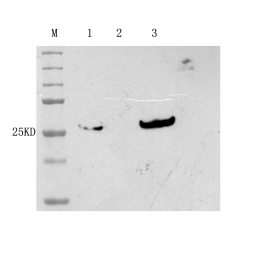


**S1 Fig. Original western blots analysis of purified HE-ORF2-tuftsin and HE-ORF2.**

Supplement: S1 Fig — Lane M, marker; lane 1, HE-ORF2; lane 2, healthy serum control; lane 3, HE-ORF2-tuftsin. (DOC) [file pone.0123400.s002.doc]

**
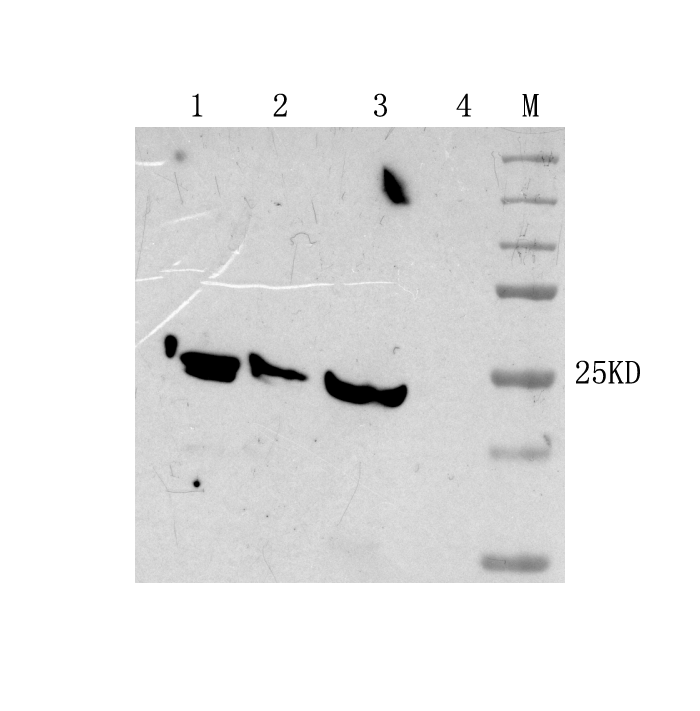
**

**S2 Fig. Original western blot analysis of purified HA-VP1-tuftsin and HA-VP1.**

Supplement: S2 Fig — Lane M, marker; lane 1 and 2, HA-VP1-tuftsin; lane 3, HA-VP1; lane 4, healthy serum control. (DOC) [file pone.0123400.s003.doc]
